# Evaluation of the Taguchi methods for the simultaneous assessment of the effects of multiple variables in the tumour microenvironment

**DOI:** 10.1186/1477-7800-1-7

**Published:** 2004-09-20

**Authors:** Hisham Morsi, Kwee L Yong, Andrew P Jewell

**Affiliations:** 1 School of Life Sciences, Kingston University, Penrhyn Road, Kingston-Upon-Thames, Surrey, KT1 2EE, UK; 2 Department of Haematology, University College London Medical School, 98 Chenies Mews, London WC1E 6HX, UK

## Abstract

**Background:**

The control of proliferation, differentiation and survival of normal and malignant cells in the tumour microenvironment is under the control of a wide range of different factors, including cell:cell interactions, cytokines, growth factors and hormonal influences. However, the ways in which these factors interact are poorly understood. In order to compare the effects of multiple variables, experimental design becomes complex and difficult to manage. We have therefore evaluated the use of a novel approach to multifactorial experimental design, the Taguchi methods, to approach this problem.

**Method:**

The Taguchi methods are widely used by quality engineering scientists to compare the effects of multiple variables, together with their interactions, with a simple and manageable experimental design. In order to evaluate these methods, we have used a simple and robust system to compare a traditional experimental design with the Taguchi Methods. The effect of G-CSF, GM-CSF, IL3 and M-CSF on daunorubicin mediated cytotoxicity in K562 cells was measured using the MTT assay.

**Results:**

Both methods demonstrated that the same combination of growth factors at the same concentrations minimised daunorubicin cytotoxicity in this assay.

**Conclusions:**

These findings demonstrate that Taguchi methods may be a valuable tool for the investigation of the interactions of multiple variables in the tumour microenvironment.

## Introduction

The control of proliferation, differentiation and survival of normal and malignant cells is under the control of a wide range of different factors. These include cell:cell interactions, immune regulatory factors, hormonal influences, and local environmental influences. However, the way in which these factors interact to regulate the dynamics of the malignant cell population are poorly understood. It is important to identify important factors and the way that they interact in order to rationalise treatment and develop new therapeutic options. However, one of the main problems is the difficulty in designing experiments to compare the effects and interactions of multiple variables. For example, a traditional experimental design to compare seven independent variables at three different concentrations each requires a large number of individual experiments (2187 experiments). The logistical and resource implications of this experimental design make these experiments very difficult to carry out. We have investigated the use of an alternative approach to experimental design, the Taguchi Methods [1]. Taguchi methods use orthogonal array distribution to design an experiment producing smaller, less costly experiments that have a high rate of reproducibility. A study involving 7 factors at 3 different concentrations can be conducted with only 18 individual experiments. Besides being efficient, the procedures for using Taguchi designs and methods are straightforward and easy to use. These methods have previously been used in PCR optimisation [2,3], baculovirus expression [4], ball and socket prosthesis design for total hip replacement surgical procedure [5], ELISA optimisation [6], and also in the evaluation of medical diagnostic tests [7,8].

We have therefore used a simple and reproducible assay, the MTT assay, to evaluate whether the Taguchi methods can be used to investigate the effect of G-CSF, GM-CSF, IL3 and M-CSF on daunorubicin mediated cytotoxicity in K562 cells.

### Taguchi Methods

Taguchi methods consist of 3 phases: designing the experiment, running and analysing, and confirming and validating the assumptions. After selecting the variables to be studied, Taguchi methods depend on distributing the factors under study in an orthogonal array, which distributes the variables (factors) in a balanced manner. Examining a typical orthogonal array (Table 1), where each factor has 2 levels or concentrations, reveals that each level has an equal number of occurrences within each column. For each column of the orthogonal distribution below, level 1 occurs four times, and level 2 occurs four times as well [1]. This idea of balance goes farther than meaning simply an equal number of levels within each column. The relationship between one column and another is arranged so that for each level within one column, each level within any other column occurs an equal number of times as well. With reference to Table 1, it can be observed that factor A is assigned to column 1, and for A at level 1, factor B is at level 1 twice and at level 2 twice. The same is true for factor A at level 2. Looking at the last column, the same relationship between factors A and G is also noted. No matter which two columns are selected, the same will be true. The ramifications of this orthogonality among columns are the basis of the statistical independence of orthogonal arrays; hence the effect of each factor can be separated from the others. Therefore, an estimation of the effect of any one particular factor tends to be accurate and reproducible because the estimated effect does not include the influence of other factors. Furthermore, each factor can be assigned a significance weight to denote its importance in affecting the end result of the experiment.

**Table 1:** Orthogonality. The relationship between one column and another is arranged so that for each level within one column, each level within any other column occurs an equal number of times as well. Factor A, at level 1 occurs 4 times and at level 2 occurs 4 times as well. This equal occurrence is true for all factors involved in any orthogonal array.

	A	B	C	D	E	F	G	Results
1	1	1	1	1	1	1	1	Y1
2	1	1	1	2	2	2	2	Y2
3	1	2	2	1	1	2	2	Y3
4	1	2	2	2	2	1	1	Y4
5	2	1	2	1	2	1	2	Y5
6	2	1	2	2	1	2	1	Y6
7	2	2	1	1	2	2	1	Y7
8	2	2	1	2	1	1	2	Y8

Each array can be identified by the form L_*A*_(B^*C*^), the subscript L, which is designated by *A*, represents the number of experiments that would be conducted using this design, B denotes the number of levels or concentrations within each column which denotes how many levels or concentrations could be investigated, while the letter *C *identifies the number of columns available within the orthogonal array which indicates how many factors or variables could be included in the experiment [1]. For example the orthogonal array L_8_(2^7^) means that 8 experimental runs are needed to investigate 7 different factors, each of which is set at 2 predetermined levels or concentrations (Table 1). The statistical independence of these arrays enables the effect of each factor to be separated from the others, the effects to be accurate and reproducible because the estimated effect does not include the effects of other factors and the interactions between these factors to be determined.

Level average analysis, as described by Taguchi [1] is one of the techniques used to explore the results of the Taguchi methods. The name derives from determining the average effect of each factor on the outcome of the experiment. The goal is to identify those factors that have the strongest effects and whether they exert their effect independently or through interacting with other factors.

The equation below illustrates the method of calculating the average effect of the experiment where Y1 is the result of the first experiment, Y2 is the result of the second experiment...etc, T is the overall average of the experiment, and n is the number of the experimental runs.



For example, in order to calculate the effect of the two concentrations of factor A, which are denoted A1 and A2, where A1 is the average effect of factor A at concentration 1, A2 is the average effect of the same factor at concentration 2.



The relative impact of each factor (ΔX) is simply the range, which could be calculated as the difference between the highest and lowest average response of each level. For example the impact of factor A on the experiment outcome is the difference between A1 & A2. (Known statistically as the range (Δ)). The effects of all factors are calculated in the same way, then arranged in a response table, and examined for those factors with the strongest effect (i.e. highest difference Δ), in order to separate them from the weak effects. The breaking point between the strong and weak effects is identified as a change in the pattern of the difference between the ranges around the median.

Besides determining the effects of the individual factors, the same technique is used to determine the strength of the impact of interactions on the product of the experiment. The calculations are performed as the previous section. In order to determine the interactions between A and B, the average result of each 2 factors combined must be determined. This is achieved through calculating the values of 4 points: A1B1, A1B2, A2B1, and A2B2, where A1B1 is the average result generated due to the interaction between concentration 1 of both factors, A1B2 is the result of the interaction between concentration 1 of factor A and concentration 2 of factor B, A2B1 represents the interaction between factor A at concentration 2 and factor B at concentration 1, while the fourth point A2B2 is the interaction between both factors at concentration 2. These 4 points are then presented graphically to show the strength or weakness of the interaction. Whether the interaction is weak, mild, or strong depends on whether the two response lines are parallel, converging or intersecting, with intersecting lines indicate a strong interaction, and parallel lines indicate no interactions. Once the strong factors and interactions have been identified, an estimate of their combined effect is calculated and the new experiment is designed according to these assumptions. An experiment is then carried out – referred here to as "confirmation run"- to validate the assumptions upon which the new experiment was based. Conducting a confirmation run and the comparison between the actual and the predicted results is necessary. If however, the confirmation results are disappointing, the planning phase must be re-evaluated and the elements that went into the experiment must be reviewed. A possible cause could be the omission of a key factor from the experiment, for example a powerful interaction was not considered. Another common cause is the setting of factor levels too close together for the experiment. In these situations, the factor is found insignificant during the analysis and is not accounted for in the validation. The confirmation run should include the best or preferred settings for mild and weak influences as well as the strong ones. However, the less influential factors are not incorporated into the prediction equation. The reasoning is that the differences in the average results may be due to experimental variation, and to incorporate their effects could result in an overestimate of the predicted results. This could lead to a disappointing confirmation run when actually the results would have validated the experiment analysis if the predicted results had not been artificially high or low.

## Methods

### Cell Culture

K562 cells were cultured in RPMI-1640 medium supplemented by 10% (v/v) foetal bovine serum, 50 μg/ml penicillin and 25 μg/ml streptomycin at 37°C in a humidified atmosphere of 5% CO_2_-95% air. Cells were plated in 96 well microtiter plates (200 μl) at a density of 3 × 10^4 ^cells/ml. Cells were co-cultured in the presence of 0.1 μg/ml daunorubicin.

### Cytokines

In all experiments K562 cells were co-incubated in the presence or absence of cytokines concentrations shown in Table 2. All cytokines were purchased from R&D Systems, UK.

**Table 2:** Concentrations of cytokines used.

		1	2
A	MCSF	100 U/ml	300 U/ml
B	IL3	10 ng/ml	50 ng/ml
C	GMCSF	10 ng/ml	50 ng/ml
D	GCSF	10/ ng/ml	50 ng/ml

### MTT Assay

50 μl of MTT (3–4,5-dimethylthiazol 2,5-diphenyl tetrazolium bromide) (5 mg/ml) was then added to each well and incubated at 37°C for 4 hours. The resulting deep blue crystals were dissolved in 0.04 N HCl Isopropyl alcohol, and the absorbance measured using a scanning multiwell spectrophotometer at dual wavelength 570–630 nm. All measurements were performed in triplicates.

The % survival was calculated as



### Classical Experimental Design

In classical experimental design the effect of each factor, each concentration and each interaction is tested independently. In order to investigate the full interactions between 4 factors each at 2 concentrations, requires 81 individual experimental conditions to be performed. This study used 49 combinations. The structure of the 49 experiments is shown in Table 3, where runs 1–16 were designed to include all the different possible combinations of all 4 cytokines together. For example, experimental run 3 was carried out after adding 100 U/ml MCSF, 50 ng/ml of both IL-3 and GMCSF, and 10 ng/ml GCSF to the medium. Runs 17–24 included the effects of each cytokine individually; two concentrations of each cytokine were tested. For example in run 17,, only MCSF was added to the medium at concentration 1 (100 U/ml), while in run 18 the same cytokine was added at concentration 2 (300 U/ml). Runs 25–48 were planned to included the different possible interactions between each 2 cytokines, for example in run 25 both MCSF (100 U/ml) and IL-3 (10 ng/ml) were added to the medium, in run 26 MCSF (100 U/ml) and IL-3 (50 ng/ml) were added, in run 27 MCSF (300 U/ml) and IL-3 (10 ng/ml) were added, and in run 28 MCSF (300 U/ml) and IL-3(50 ng/ml) were added. Finally, experimental run 49 was carried out without adding any cytokines to the medium.

**Table 3:** The whole set of the 49 experiments carried out. Runs 1–16 included all possible combinations of all cytokines together (see text above). In runs 17–24 individual cytokine were added to the medium, two concentrations of each cytokine was tested. For example in run 17 MCSF was added to the medium at concentration 1 (100 U/ml), while in run 18 the same cytokine was added at concentration 2 (300 U/ml). Runs 25 – 48 included the different possible interactions between each 2 cytokines, for example in run 25 both MCSF (100 U/ml) and IL-3 (10 ng/ml) were added, in run 26 MCSF (100 U/ml) and IL-3 (50 ng/ml) were added, in run 27 MCSF (300 U/ml) and IL-3 (10 ng/ml) were added, and in run 28 MCSF (300 U/ml) and IL-3 (50 ng/ml) were added. Experimental run 49 was carried out without adding any cytokines to the medium. All experimental runs were done in triplicate and repeated three times.

	A MCSF	B IL-3	C GMCSF	D GCSF
1	1	1	1	1
2	1	1	2	2
3	1	2	1	2
4	1	2	2	1
5	2	1	1	2
6	2	1	2	1
7	2	2	1	1
8	2	2	2	2
9	1	1	1	2
10	1	2	1	1
11	1	1	2	1
12	1	2	2	2
13	2	1	1	1
14	2	2	2	1
15	2	1	2	2
16	2	2	1	2
17	1	0	0	0
18	2	0	0	0
19	0	1	0	0
20	0	2	0	0
21	0	0	1	0
22	0	0	2	0
23	0	0	0	1
24	0	0	0	2
25	1	1	0	0
26	1	2	0	0
27	2	1	0	0
28	2	2	0	0
29	1	0	1	0
30	1	0	2	0
31	2	0	1	0
32	2	0	2	0
33	1	0	0	1
34	1	0	0	2
35	2	0	0	1
36	2	0	0	2
37	0	1	1	0
38	0	1	2	0
39	0	2	1	0
40	0	2	2	0
41	0	1	0	1
42	0	1	0	2
43	0	2	0	1
44	0	2	0	2
45	0	0	1	1
46	0	0	1	2
47	0	0	2	1
48	0	0	2	2
49	0	0	0	0

### Taguchi Design L_8_(2^7^)

In order to evaluate the performance of the Taguchi methods, eight experimental runs were carried out employing the orthogonal array L_8_(2^7^) to investigate the effect of 4 cytokines on the survival of K562 leukaemic cells. This array accommodated 4 factors MCSF, IL-3, GMCSF and GCSF. The interaction between MCSF and the other cytokines was inserted into the array. As the 3^rd ^column was used to examine the interaction between MCSF and IL-3, columns 5 and 6 were used to assess the interaction between the same cytokine and GMCSF and GCSF respectively (Table 4). All factors in this design were set at 10 and 50 ng/ml except MCSF, which was set at 100 and 300 U/ml.

**Table 4:** Taguchi method L_8_(2^7^). This array accommodated 4 different factors (MCSF, IL-3, GMCSF, and GCSF) each at 2 different concentrations (see above). 8 experimental runs were carried out according to the combination of factors in the array, for example, in experimental run 1 the MTT assay was carried out after mixing the cells with 100 U/ml MCSF, 10 ng/ml IL-3, 10 ng/ml GMCSF, and 10 ng/ml GCSF. The interaction between MCSF and the other three factors (IL-3, GMCSF and GCSF) was studied in this array.

	A MCSF	B IL3	AxB	C GMCSF	AxC	AxD	D GCSF
1	1	1	1	1	1	1	1
2	1	1	1	2	2	2	2
3	1	2	2	1	1	2	2
4	1	2	2	2	2	1	1
5	2	1	2	1	1	1	2
6	2	1	2	2	2	2	1
7	2	2	1	1	1	2	1
8	2	2	1	2	2	1	2

## Results and Discussion

### Results of the classical design

**Figure 1:**
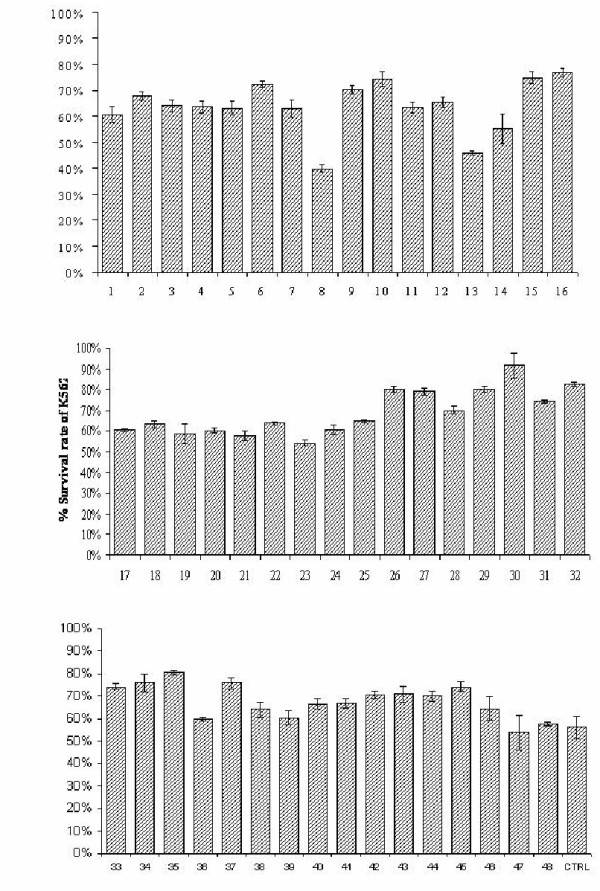
Experimental runs 1–16 show the different survival rates of K562 cells as a result of culturing the cells in medium enriched by different combinations of the 4 cytokines (GMCSF, MCSF, IL-3, and GCSF). The maximum cytotoxicity of daunorubicin was observed as a result of the addition of 300 U/ml of MCSF, and 50 ng/ml of the other 3 cytokines (experimental run 3). The maximum survival of the cells was observed when the concentration of GMCSF in this mixture was reduced to 10 ng/ml (experimental run 16). Experiments 17 – 48 suggested that MCSF interacts with the 3 other factors to affect daunorubicin cytotoxicity. This could be seen by comparing the effect of the individual factors (runs 17 – 24) with the effects of the addition of two factors simultaneously. For example, experimental run 30 shows the concurrent effect of both MCSF (100 U/ml) and GMCSF (50 ng/ml) that resulted in a survival that was significantly higher than that caused by any of the two factors alone (runs 17, 18, 21 & 22). Experimental run 26 also represents the combined effects of MCSF (100 U/ml) and IL-3 (50 ng/ml), which resulted in a survival that was higher than the resulting survival of any of the two factors individually. Run 36; on the other hand, represents the increase in daunorubicin cytotoxicity as a result of the simultaneous addition of MCSF (300 U/ml) and GCSF (50 ng/ml). All these experimental runs were done in triplicate and repeated 3 times, the results are expressed as mean ± SE.

The results of the 49 experimental conditions are shown in Figure 1. Columns 1–24 represent the simultaneous combinations of the 4 cytokines. The toxicity of daunorubicin was maximally enhanced by the addition of the four cytokines to the medium i.e. MCSF at a concentration of 300 U/ml, IL-3 at a concentration of 50 ng/ml, GMCSF at a concentration of 50 ng/ml, and GCSF at a concentration of 50 ng/ml. This resulted in a highly significant reduction of malignant cell survival, from 69% (the survival rate for the control cells) to 39% (P <0.001).

The survival of K562 cells, using the 4 cytokines simultaneously was maximally enhanced by the addition of 300 U/ml MCSF, 50 ng/ml IL-3, 50 ng/ml GMCSF, and 50 ng/ml GCSF. A significant improvement in cell survival from 69% to 76% (P 0.02) was observed

### Taguchi analysis

The results of the 8 experiments of Taguchi's L_8 _series (Table 5), were analysed in order to determine the mean effect of each factor.

**Table 5:** the results of L_8_(2^7^). Each experimental run was done in triplicate and repeated 3 times, the mean values were calculated and the results were expressed as mean ± SE. Y1 (experimental run 1), for example, = the mean survival of the cells at 100 U/ml MCSF, 10 ng/ml IL-3, 10 ng/ml GMCSF, and 10 ng/ml GCSF. The overall average of the experiment (T) was calculated as the mean of all eight experimental runs.

	% survival
Y1	60.71 ± 5.9
Y2	67.83 ± 1.9
Y3	64.01 ± 1.1
Y4	63.51 ± 2.0
Y5	62.97 ± 4.3
Y6	72.27 ± 4.3
Y7	62.86 ± 1.1
Y8	39.84 ± 1.9
T	61.75

For example the mean effect of MCSF when added to the medium at a concentration of 100 U/ml was computed as follows:



When MCSF was added to the medium at a concentration of 300 U/ml the mean effect was:



These computed values were used to construct a response table (Table 6). This shows the average mean effect for each factor and the relative impact or range of each factor on the variability of the mean. This showed that the following concentrations were associated with lower survival of the malignant cells; 300 U/ml MCSF was associated with the survival of 59.4%, 50 ng/ml of IL-3 was associated with 57.5 % survival rate, 50 ng/m GMCSF resulted in the survival of 60.8% of the cells, and finally 50 ng/ml of GCSF was associated with 58.6% survival rate.

**Table 6:** Response table for the orthogonal array L_8 _(2^7^). The average effect of each factor level is calculated and the range of effect of each factor is calculated as the difference between the two readings. The range of MCSF effect, for example = 64.02-59.48 = 4.53, the higher the range the stronger the effect of the factor. In this experiment the interaction between MCSF and GCSF had the strongest effect on the survival of cells.

	A = MCSF	B = IL3	AxB	C = GMCSF	AxC	AxD	D = GCSF
1	64.02%	65.95%	57.81%	62.64%	59.21%	56.76%	64.84%
2	59.48%	57.55%	65.69%	60.86%	64.29%	66.74%	58.66%
Δ	4.53	8.39	7.88	1.77	5.08	9.98	6.17
		2	3			1	4

In order to determine the strong effects and separate them from the weak ones, the response table was rearranged by ranking the factors in order from the largest difference to the smallest as it can be seen in Table 7. In this study the interaction between MCSF and GCSF (AXD) has the greatest effect on the survival of K562 cells. IL-3 (B) is next with a difference between the Δ's of 1.594. The interaction between MCSF and IL-3 (AXB) was next followed by GCSF (D). The difference between ΔB and ΔAXB is 0.507 (8.391-7.884 = 0.507), if we continue farther to factor D the difference in effects jumps to 1.711 (7.884-6.173 = 1.711). Therefore this point would be considered as the breaking point. The factors to its left (AXD, B, and AXB) are the important factors i.e. MCSF, IL-3, and GCSF. MCSF exerts strong effects through interacting with both IL-3 and GCSF.

**Table 7:** Descending rearrangement of the response table according to strong and weak effects. The response table was rearranged according to the Δs, and the difference between the Δs was calculated and then scanned to determine the break point, which was identified as a change in the pattern of the difference between the Δs around the median. The strong factors would be on the left hand side of the break point, marked in this table in bold.

AxD	B = IL3	AxB	D = GCSF	AxC	A = MCSF	C = GMCSF
9.985%	8.391%	7.884%	6.173%	5.088%	4.533%	1.774%
	**1.594**	**0.507**	1.711	1.085	0.555	2.759

In order to study each interaction incorporated into this design, an interaction matrix for each interaction was constructed as described above; hence a 2 × 2 matrix was constructed for each interaction. For example to construct an interaction matrix for MCSF and GCSF (AXD), four points were computed A1D1, A1D2, A2D1, and A2D2 (Table 8).

**Table 8:** Interaction matrix AxD. The average effect of the four points of this interaction matrix on the survival of K562 cells. The preferred setting of this interaction that would maximise the cytotoxicity of daunorubicin is A2D2 i.e. 300 U/ml of MCSF and 50 ng/ml of GCSF. This combination would result in a survival of 51.67% of the cells.

	D1	D2
A1	62.11%	65.92%
A2	67.56%	**51.67%**

A1D1 is the average result of the combined effect of MCSF at a concentration 100 U/ml and GCSF at a concentration of 10 ng/ml.

**Figure 2:**
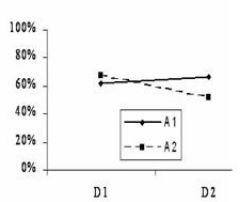
Graphical presentation of interaction between AxD (MCSF and GCSF). Intersecting lines of this graph indicate strong interaction. A2D2 is the preferred point on the graph i.e. the combination of these two factors to produce maximum daunorubicin cytotoxicity.

The interaction matrix AXD was then represented graphically (Fig 2) which showed intersecting lines indicating a strong interaction. Both table 8 and fig 2 were further studied to decide which combination suites the desired outcome of the experiment; hence for the smaller the better outcome, it is clear that 51.67% is the lowest survival value in this matrix (Table 8), i.e. A2D2 (MCSF at a concentration of 300 U/ml and GCSF at a concentration of 50 ng/ml) is associated with the lowest survival rate of the malignant cells. The interaction between MCSF and the two other factors was further studied and the preferred combinations for both interactions were A2B2 i.e. 300 U/ml MCSF and 50 ng/ml IL-3, which was associated with 51.35% survival rate, and A2C2, i.e. MCSF at concentration 2 (300 U/ml) and 50 ng/ml GMCSF, which was associated with 56.05% survival. The preferred settings suggested by this analysis to optimise the cytotoxicity of daunorubicin was combining the following factors A2B2C2D2 i.e. MCSF at a concentration of 300 U/ml, IL-3 at a concentration of 50 ng/ml GMCSF at a concentration of 50 ng/ml, and GCSF at a concentration of 50 ng/ml.

An estimate of the predicted response (μ) based on the selected levels was then computed. The calculations were based on the overall average value (T) and the effect that each of the recommended levels of the strong factors and interactions has on the overall average.

μ = T+(A2D2 - T)+(B2 - T)+(A2C2 - T)+(A2B2 - T)-(A2 - T)-(A2 - T)-(A2 - T)-(B2 -T)-(C2-T).

The reason for subtracting the individual effects of factors A, B, and C from the effects of A2B2 is that A2B2 is comprised of the effects of factor A, factor B and the interaction itself. Unless the effects of the two factors are subtracted these strong effects would be included twice and resulting in an overestimation of the predicted result.

The predicted survival derived from the above prediction equation was 43%. A confirmation run that produces a %survival close to 43%would validate the assumptions of this Taguchi method. The actual confirmation run, in fact, resulted in 39.84% survival rate indicating the success of the Taguchi experiment.

Further calculations were performed to determine whether the outcome of other combinations could be predicted from the Taguchi experiment and confirmed by analysis. The results are shown in Table 9 and show a close approximation in each case.

**Table 9:** Further comparison of the predicted values from the Taguchi Methods, and the result produced by experimental analysis.

	Prediction	Analysis
Experimental run 9	66.10%	70.35%
Experimental run 10	71.93%	74.38%
Experimental run 11	66.14%	63.36%
Experimental run 12	67.65%	65.36%
Experimental run 13	48.90%	45.69%
Experimental run 14	51.46%	55.22%
Experimental run 15	63.65%	74.88%
Experimental run 16	72.19%	76.74%

## Conclusion

The aim of this study was to evaluate the ability of the Taguchi methods to investigate the effects of several factors simultaneously on the death and/or survival of the malignant cells, and to compare this strategy against a traditional full experimental design.

A major finding of the study was that the Taguchi methods predicted the combination of factors that results in the lowest survival of the malignant cells. This agreed with the conclusions of the full experimental design but required only eight individual experiments to pinpoint this combination. However, it must be stressed that the Taguchi methods are not intended to be a replacement for traditional experimental design, but if used as a complimentary strategy can make analysis of complex interactions feasible and practical. In this study, for example, eight individual experiments produced a testable combination which required 49 individual experiments to produce in the traditional experimental design. In more complex systems, only Taguchi methods become feasible. For example, to study 13 factors at 3 different combinations would require 1,594,323 individual experiments at a cost for re agents alone of over 27 million pounds. If Taguchi methods are used, this can be reduced to just 27 individual experiments at a cost of under 500 pounds.

We have described a novel experimental approach to studying the interactions of several factors on the cytotoxicity of malignant cells. We show that the method is effective in the determination of the optimum conditions, even in the presence of multiple interactions. We anticipate that this experimental strategy will have many applications in the investigation of complex interactions. For example we have used this strategy to model the complex testicular microenvironment and the ability to support the survival of acute lymphoblastic leukaemia cells (manuscript in preparation). These sort of interactions are common in the survival of malignant cells *in vivo*, and we propose that the Taguchi methods may be a useful strategy to understand these interactions *in vitro*, and to help devise and implement new therapeutic strategies.

## Competing interests

None declared.

## Authors' contributions

HM designed the Taguchi assay, carried out cell survival assays, and drafted the manuscript. KLY participated in the design and coordination, and produced the final manuscript. APJ conceived the study and participated in its design and coordination. All authors read and approved the final manuscript.
